# Life History Responses of Four Invasive Crayfish Species Under Prolonged Suboptimal Temperatures

**DOI:** 10.1093/icb/icag014

**Published:** 2026-03-23

**Authors:** Antonín Kouba, Koushik Das, Wei Guo, Kateřina Marková, Lukáš Veselý, Francisco J Oficialdegui, Boris Lipták, Jan Kubec, Anna Koubová, Martin Bláha, Hamid Niksirat, Jiří Patoka, András Weiperth, Phillip J Haubrock, Miloš Buřič

**Affiliations:** Faculty of Fisheries and Protection of Waters, South Bohemian Research Center of Aquaculture and Biodiversity of Hydrocenoses, University of South Bohemia in České Budějovice, Zátiší, 728/II, 389 01 Vodňany, Czech Republic; Faculty of Fisheries and Protection of Waters, South Bohemian Research Center of Aquaculture and Biodiversity of Hydrocenoses, University of South Bohemia in České Budějovice, Zátiší, 728/II, 389 01 Vodňany, Czech Republic; College of Biosystems Engineering and Food Science, Zhejiang University, 310000 Hangzhou, China; Faculty of Fisheries and Protection of Waters, South Bohemian Research Center of Aquaculture and Biodiversity of Hydrocenoses, University of South Bohemia in České Budějovice, Zátiší, 728/II, 389 01 Vodňany, Czech Republic; Xinjiang Key Laboratory for Ecological Adaptation and Evolution of Extreme Environment Organisms, College of Life Sciences, Xinjiang Agricultural University, Nongda East Road No. 311, Shayibak District, Urumqi 830000, China; Faculty of Fisheries and Protection of Waters, South Bohemian Research Center of Aquaculture and Biodiversity of Hydrocenoses, University of South Bohemia in České Budějovice, Zátiší, 728/II, 389 01 Vodňany, Czech Republic; Faculty of Fisheries and Protection of Waters, South Bohemian Research Center of Aquaculture and Biodiversity of Hydrocenoses, University of South Bohemia in České Budějovice, Zátiší, 728/II, 389 01 Vodňany, Czech Republic; Faculty of Fisheries and Protection of Waters, South Bohemian Research Center of Aquaculture and Biodiversity of Hydrocenoses, University of South Bohemia in České Budějovice, Zátiší, 728/II, 389 01 Vodňany, Czech Republic; Department of Conservation Biology and Global Change, Doñana Biological Station (CSIC), C/Americo Vespucio 26, 41092 Seville, Spain; Faculty of Fisheries and Protection of Waters, South Bohemian Research Center of Aquaculture and Biodiversity of Hydrocenoses, University of South Bohemia in České Budějovice, Zátiší, 728/II, 389 01 Vodňany, Czech Republic; Slovak Environment Agency, Tajovského 28, 975 90 Banská Bystrica, Slovak Republic; Faculty of Fisheries and Protection of Waters, South Bohemian Research Center of Aquaculture and Biodiversity of Hydrocenoses, University of South Bohemia in České Budějovice, Zátiší, 728/II, 389 01 Vodňany, Czech Republic; Faculty of Fisheries and Protection of Waters, South Bohemian Research Center of Aquaculture and Biodiversity of Hydrocenoses, University of South Bohemia in České Budějovice, Zátiší, 728/II, 389 01 Vodňany, Czech Republic; Faculty of Fisheries and Protection of Waters, South Bohemian Research Center of Aquaculture and Biodiversity of Hydrocenoses, University of South Bohemia in České Budějovice, Zátiší, 728/II, 389 01 Vodňany, Czech Republic; Faculty of Fisheries and Protection of Waters, South Bohemian Research Center of Aquaculture and Biodiversity of Hydrocenoses, University of South Bohemia in České Budějovice, Zátiší, 728/II, 389 01 Vodňany, Czech Republic; Department of Zoology and Fisheries, Faculty of Agrobiology, Food and Natural Resources, Czech University of Life Sciences Prague, Kamýcká 129, 165 00 Prague-Suchdol, Czech Republic; Department of Preschool and Primary Education, Faculty of Education, Jan Evangelista Purkyně University in Ústí nad Labem, České mládeže 8, 400 01 Ústí nad Labem,Czech Republic; Department of Biology, Faculty of Science, Humanities and Education, Technical University of Liberec, Studentská 1402/2, 461 17 Liberec, Czech Republic; Department of Systematic Zoology and Ecology, Institute of Biology, ELTE Eötvös Loránd University, Pázmány Péter ave 1/C, H-1117 Budapest, Hungary; Dr. Puky Miklós Toad Action Group, Endrődi Sándor street,85/A, H-1026 Budapest, Hungary; Faculty of Fisheries and Protection of Waters, South Bohemian Research Center of Aquaculture and Biodiversity of Hydrocenoses, University of South Bohemia in České Budějovice, Zátiší, 728/II, 389 01 Vodňany, Czech Republic; Department of Life and Environmental Sciences, Bournemouth University, Poole, Dorset, Talbot Campus, Fern Barrow, Poole, Dorset BH12 5BB, UK; Faculty of Fisheries and Protection of Waters, South Bohemian Research Center of Aquaculture and Biodiversity of Hydrocenoses, University of South Bohemia in České Budějovice, Zátiší, 728/II, 389 01 Vodňany, Czech Republic

## Abstract

Biological invasions are strongly shaped by temperature, especially in poikilothermic organisms, where thermal regimes influence life-history traits, thereby determining both their competitive potential and geographic distribution. However, comparative evidence on how suboptimal thermal conditions modulate interactions among co-occurring invasive species remains scarce. We experimentally compared the growth, survival, and reproductive performance of the invasive parthenogenetic marbled crayfish *Procambarus virginalis* with three widespread North American crayfish invaders in Europe: the spiny-cheek crayfish *Faxonius limosus*, the signal crayfish *Pacifastacus leniusculus*, and the red swamp crayfish *Procambarus clarkii*. Experiments were conducted under prolonged suboptimal temperature conditions (∼16°C over 45 weeks), followed by a short-term temperature increase (∼20°C). Across three independent laboratory trials, we assessed species performance in single-species and mixed-species stocks. Despite reduced absolute growth rates at low temperature, marbled crayfish rapidly compensated for their initially smaller size and outperformed spiny-cheek crayfish in growth and survival. In contrast, marbled crayfish were consistently suppressed when co-occurring with the larger and more aggressive red swamp crayfish, whereas interactions with signal crayfish resulted in temporary growth advantages but ultimately size convergence. Survival patterns reflected a combination of size asymmetries, behavioral dominance, and intraspecific aggression, with marbled crayfish exhibiting notably high survival in single-species stocks across all trials. Reproductive development was strongly temperature-constrained. While marbled crayfish readily formed glair glands and ovulated eggs at 16°C, successful hatching occurred only after the temperature was raised. Our results demonstrate that suboptimal thermal conditions do not eliminate competitive asymmetries among invasive crayfish but instead reshape invasion outcomes in species-specific ways. These findings highlight the marbled crayfish’s capacity to persist and interact competitively even in colder environments, with important implications for invasion dynamics under ongoing climate change.

## Introduction

The introduction of species beyond their native ranges can result in the establishment of self-sustaining non-native populations that spread and generate ecological, economic, or sociocultural impacts (Haubrock et al. 2025a, b), thereby qualifying them as invasive non-native species (see Soto et al. 2024, for further reference). Freshwater ecosystems are particularly vulnerable to biological invasions (Sayer et al. 2025), and numerous invasive taxa not only impact native communities but also interact competitively with other non-native species (Gherardi et al. 2011; Havel et al. 2015). Among these, freshwater crayfish rank among the most damaging invaders globally (Lodge et al. 2012).

Across taxa, invasion success is shaped by environmental filters that interact with species’ thermal performance and life–history schedules (Blackburn et al. 2014). In poikilotherms in particular, temperature scales core processes, such as metabolism, feeding, growth, aggression, and reproduction (Angilletta Jr 2009), thereby modulating establishment, spread, and the strength and direction of biotic interactions (O’Connor et al. 2011). This framework predicts that both suboptimal and elevated temperatures can re–rank competitive hierarchies among co–occurring invaders when rivals differ in thermal tolerance, plasticity, or behavioral dominance (Kordas et al. 2011; Urban et al. 2016).

Within this broader context, freshwater crayfish provide a well-established model group for studying temperature-sensitive invasion dynamics. Europe is disproportionately affected by non-native crayfish of North American origin, which frequently displace native species through competition and transmission of the crayfish plague pathogen *Aphanomyces astaci*, to which European crayfish are highly susceptible (Ion et al. 2024; Mojžišová et al. 2024; Oficialdegui et al. 2025). Three long-established invaders (the spiny-cheek crayfish *Faxonius limosus*, signal crayfish *Pacifastacus leniusculus*, and red swamp crayfish *Procambarus clarkii*) are now widespread (Kouba et al. 2014). More recently, the parthenogenetic marbled crayfish, *Procambarus virginalis*, has expanded rapidly across the continent, largely through repeated pet-trade releases (Patoka et al. 2016; Sánchez et al. 2024). Continued introductions increase the likelihood of co-occurrence among invasive taxa, creating conditions for competitive reordering under varying environmental constraints (Weiperth et al. 2020; Balzani et al. 2025).

This trend towards over-invasion scenarios raises critical questions about species’ competitive performance under varying environmental conditions, which can be optimal for some species but suboptimal for others (Hudina et al. 2011; Kouba et al. 2016; Balzani et al. 2025). Consequently, understanding how environmental factors, such as temperature, mediate establishment success, range expansion, and interspecific interactions is essential for predicting relative abundance patterns and long-term persistence in invaded communities (Carvalho et al. 2022; O’Hea Miller et al. 2024). Species such as the marbled crayfish and red swamp crayfish are typically regarded as warm-water taxa, although both exhibit substantial tolerance to low temperatures, as demonstrated by their persistence in relatively cold climates (Veselý et al. 2015; Haubrock et al. 2019; Hamr 2024; Mengal et al. 2026). However, their introductions have often occurred in thermally influenced habitats, including naturally warm waters or sites affected by thermal pollution in temperate regions (Ercoli et al. 2019; Bláha et al. 2022). Besides crayfish, diverse warm-water taxa, including aquatic plants, molluscs, shrimps, fish, and turtles, can use these habitats as a source for outward spread into surrounding habitats and for gradual acclimation to temperate climate conditions (Lőkkös et al. 2016; Lipták et al. 2018; Šajna et al. 2023; Takács et al. 2025). Nevertheless, releases associated with irresponsible or unaware pet ownership can occur virtually anywhere, including environments with suboptimal thermal conditions, as evidenced by records from Poland, Sweden, and the North American Great Lakes region (Bohman et al. 2013; Maciaszek et al. 2022; Hamr 2024).

To assess whether suboptimal thermal conditions act as a selective filter that reshapes competitive hierarchies among co-occurring non-native crayfish, we conducted controlled comparative experiments focusing on their growth, survival, and reproduction. We examined the performance of marbled crayfish relative to spiny-cheek crayfish, signal crayfish, and red swamp crayfish under suboptimal temperature conditions (∼16°C) across three independent trials. Using invasive crayfish as a model system, these experiments provide mechanistic insight into how temperature constrains invader performance and modulates interspecific competitive outcomes. Because the same thermal and behavioral mechanisms govern many freshwater invaders, our comparative, low–temperature approach offers a transferable framework for anticipating invasion trajectories under suboptimal thermal regimes. This knowledge is essential for improving risk–assessment frameworks and anticipating the dynamics of current and emerging crayfish invasions in temperate freshwater systems under present–day climatic conditions and future scenarios.

## Materials and methods

### Animal origin and juvenile selection

Egg-bearing females of spiny-cheek crayfish and signal crayfish were collected from established feral populations in the Czech Republic, specifically from the Blanice river in Protivín (49.186°N, 14.221°E) and Křesanovský brook in Vimperk (49.059°N, 13.761°E), respectively. Females were transported to the laboratory several weeks before hatching and were gradually acclimated to the experimental temperature regime (i.e., ∼16°C). The other two species originated from closed laboratory cultures maintained at the Research Institute of Fish Culture and Hydrobiology in Vodňany and were derived from locally sourced pet-trade individuals. The red swamp crayfish has not yet been established in the Czech Republic (Kouba et al. 2014). At the time of the experiments (conducted from 2019 to 2021), existing marbled crayfish populations (Patoka et al. 2016) were insufficient to obtain the required number of egg-bearing females. Although logistically demanding, only juveniles that first reached independence (accompanied by the onset of exogenous feeding) on the same day were selected and matched across species to ensure strict comparability of performance. Signal crayfish juveniles reach independence at the second developmental stage, whereas juveniles of the remaining species were used at the third developmental stage (Kawai and Kouba 2022).

### Experimental design

To better understand the performance of juvenile marbled crayfish relative to the abovementioned invasive species under suboptimal temperature conditions, we conducted three independent laboratory experiments comparing growth, survival, and reproduction. Each trial included marbled crayfish and one of the three additional species, and comprised three experimental groups: two monocultures (one per paired species) stocked at an initial density of 16 juveniles per aquarium (hereafter referred to as “single-species” stocks), and one communal stock with the same total density but an equal species ratio (1:1; eight juveniles of each species; hereafter referred to as “mixed-species” stock). We replicated each experimental combination five times, resulting in 15 aquaria per trial and 45 aquaria in total across all trials.

In our previous study (Kouba et al. 2021), conducted under near-optimal warm conditions (∼22°C), marbled crayfish extruded and carried pleopodal eggs for the first time between weeks 14 and 16 of culture (∼16–18 weeks of age, including the early postembryonic development of juveniles; Guo et al. 2019). Based on this work, the present study focused on species performance under suboptimal temperature conditions (∼16°C). Seitz et al. (2005) reported that marbled crayfish cease reproduction at temperatures of 15°C or below. As growth and maturation are strongly temperature-dependent, with lower temperatures slowing growth and delaying maturation, the experimental period under the selected temperature regime was extended to 45 weeks. During this period, marbled crayfish ovulated; however, successful hatching did not occur (see Results for details). Consequently, the temperature was increased to ∼20°C for an additional 9 weeks (until week 54) in the experimental groups involving marbled crayfish and red swamp crayfish, the only species that consistently formed glair glands and ovulated eggs within the initial 45-week period. The spiny-cheek crayfish and signal crayfish stocks were terminated at that point, while their corresponding marbled crayfish stocks continued. The light: dark photoperiod was maintained at 12:12 h.

Although each species has a distinct thermal optimum, 16°C constitutes a biologically suboptimal condition for all four taxa, enabling meaningful performance comparisons without introducing confounding species–specific temperature treatments. It can be more restrictive for the warm–water red swamp crayfish and marbled crayfish than the cold–adapted crayfish species (Westhoff and Rosenberger 2016). Even for the latter group, natural summer temperatures commonly exceed 20°C (Bohman et al. 2016), indicating that 16°C lies below the typical thermal range experienced by all species. We therefore applied a single, uniform temperature regime to ensure direct interspecific comparisons under identical environmental constraints.

### Culturing conditions and feeding

Unsexed juveniles were randomly stocked into a laboratory recirculating rearing system consisting of glass aquaria (37 cm width × 55 cm length × 31.5 cm height; usable volume 55 L), which served as culture units. To minimize aggression and cannibalism, shelters were provided in the form of a fired clay brick (6.5 × 28.5 × 13.5 cm) containing 39 cross holes (26 and 13 holes with cross-sectional profiles of 1 × 3 cm and 1 × 1 cm, respectively) placed in each aquarium (Kouba et al. 2011). Consequently, the number of available individual shelters exceeded the initial number of stocked juveniles by more than twofold.

As crayfish grew during the experiment, two blocks of joined polypropylene tubes, each comprising five tubes (length 10 cm, inner diameter 35 mm), were added as larger shelters after 9 weeks of culture. Each block consisted of three longitudinally joined tubes forming the base, with two additional tubes arranged pyramidally in a second layer (Veselý et al. 2017).

In all trials, crayfish were fed *ad libitum* once daily with defrosted brine shrimp (*Artemia* sp.) nauplii and chironomid larvae during the first 6 weeks to minimize resource limitation and isolate temperature and interspecific interaction effects from density-dependent food competition. Thereafter, the diet was changed to defrosted chironomid larvae supplemented with commercial pellets (Granugreen, Sera, Heinsberg, Germany). Aquaria were cleaned three times per week (Monday, Wednesday, and Friday), and the system was replenished with drinking tap water as required.

To minimise handling stress, individual weights (after removal of excess surface water using absorbent tissue paper) were recorded every 3 weeks using an analytical balance (Kern and Sohn GmbH, Balingen, Germany) for a total of 45 weeks. As individuals grew and matured, they were also sexed and examined for the presence of glair glands and attached eggs (for details see Das et al. 2025). For marbled crayfish and red swamp crayfish, the only species exhibiting consistent formation of glair glands and egg deposition within the initial 45-week period, reproductive parameters were monitored under increased water temperature of ∼20°C for another 9 weeks.

Water temperature (T,°C), dissolved oxygen (DO, mg L^−1^; measured using Oxi 315i, WTW GmbH, Weilheim, Germany), and pH (measured using a pH 315i, WTW GmbH, Weilheim, Germany) were recorded daily and remained generally stable throughout the experiment (Table 1).

**Table 1:** Basic water quality parameters (temperature,°C; dissolved oxygen, DO, mg L⁻¹; and pH) were recorded in each trial comparing marbled crayfish *P. virginalis* with comparator species (spiny-cheek crayfish *F. limosus*, signal crayfish *P. leniusculus*, or red swamp crayfish *P. clarkii*) under the respective temperature regimes.

	First 45 weeks						Additional 9 weeks					
	T		DO		pH		T		DO		pH	
Trial	Mean	SD	Mean	SD	Mean	SD	Mean	SD	Mean	SD	Mean	SD
*F. limosus*	16.1	0.3	8.7	0.7	7.7	0.2	20.2	0.6	7.8	0.6	7.8	0.1
*P. leniusculus*	16.0	0.3	8.5	0.6	7.7	0.3	20.0	0.9	8.1	0.6	7.8	0.3
*P. clarkii*	16.1	0.6	9.3	0.8	7.3	0.4	20.2	0.4	7.9	0.5	7.2	0.2

*Note:* Data are presented as means with their respective SDs.

### Statistical analyses

Weight data were evaluated for normality and homogeneity of variances using the Kolmogorov–Smirnov and Levene’s tests, respectively. Because several groups violated test assumptions, comparisons of weights between single- and mixed-stock treatments were conducted using non-parametric Kruskal–Wallis tests, followed by post hoc multiple comparisons of mean ranks. Sex-related weight differences in sexually reproducing species were assessed at week 45 using nonparametric Mann–Whitney U tests for both single- and mixed-stock treatments. Nonparametric survival analyses were performed using the Kaplan–Meier method implemented in the *survival* package in R (Therneau and Grambsch 2000) to test for significant differences among specific pairwise assemblages. Additionally, survival rates, calculated as % survival from the initial stocking densities, were arc-sine transformed and assessed as weight data. Statistical analyses other than survival analyses were conducted using Statistica 14.0.0.15 for Windows (TIBCO Software Inc). Data are presented as mean and their respective SD unless specified otherwise, and results were considered statistically significant at *P* < 0.05.

## Results

### Growth

The initial body mass of juveniles at the onset of exogenous feeding varied substantially among species across all trials. Based on a subsample of individuals not subsequently used in the experiment, spiny-cheek crayfish juveniles were almost twice as heavy as marbled crayfish (mean = 11.0, SD = 1.5 mg vs. mean = 5.7, SD = 0.7 mg; *n* = 20 per species). A similar pattern was observed in the comparison with red swamp crayfish (mean = 11.8, SD = 1.3 mg vs. mean = 5.1, SD = 0.3 mg; *n* = 10 for both species). The greatest disparity occurred in the signal crayfish trial, in which juveniles were more than four times heavier than marbled crayfish (mean = 24.2, SD = 2.2 mg vs. mean = 5.3, SD = 0.6 mg; *n* = 10 for both species).

**Fig. 1:**
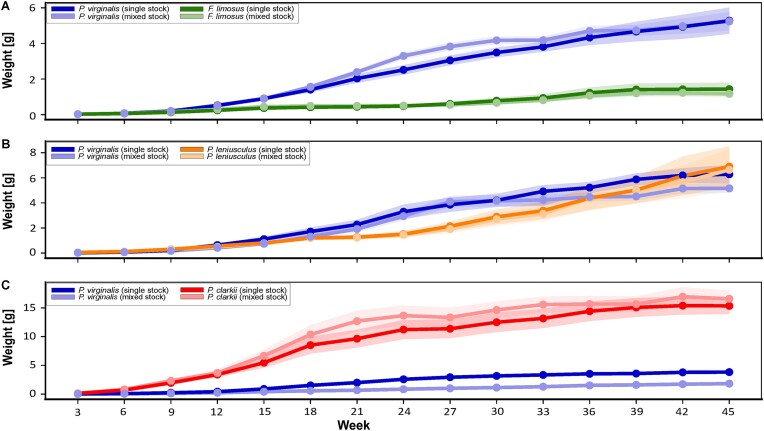
Mean body weight (g) over time of marbled crayfish *Procambarus virginalis* in experimental trials with (A) spiny-cheek crayfish *Faxonius limosus*, (B) signal crayfish *Pacifastacus leniusculus*, and (C) red swamp crayfish *Procambarus clarkii* under single-species and mixed-species stocking conditions. In each panel, darker shades represent single-species stocks and lighter shades represent mixed-species stocks. Points indicate weekly means, and shaded areas represent 95% confidence intervals. For data presented as mean and SD, see Fig. S1; for individual measurements, see Fig. S2.

All species exhibited pronounced but distinct growth trajectories (Fig. 1 and Fig. S1). Despite their initially smaller size, marbled crayfish reached body masses comparable to those of the larger spiny-cheek crayfish within 3 weeks of culture. From the sixth week onwards, marbled crayfish exceeded spiny-cheek crayfish in size in both single-species and mixed-species stocks (Fig. S1). No statistically significant differences in body mass were detected between single- and mixed-species stocks within species. After 45 weeks, only a single-stocked spiny-cheek crayfish exceeded 5 g in body mass, whereas several individuals surpassed 10 g in the marbled crayfish in both single- and mixed-stocks (Fig. S2). Spiny-cheek crayfish males and females were similarly sized in both types of stock after 45 weeks (Fig. S3).

Signal crayfish juveniles remained significantly larger for at least the first 9 weeks of culture (Fig. 1 and Fig. S1). However, marbled crayfish exhibited greater growth during this period. Body masses of both species converged between weeks 12 and 18, after which marbled crayfish prevailed in size (weeks 21–30). With the onset of reproductive development (see below), growth increments in marbled crayfish declined, and body sizes of both species became largely comparable again, with no significant differences detected at weeks 42 and 45 (Fig. S1). Despite higher growth rates and larger mean body mass of marbled crayfish over most of the experimental period, signal crayfish ultimately achieved greater individual sizes. After 45 weeks, only three marbled crayfish individuals exceeded 10 g (maximum 16.64 g) in single-species stocks, whereas twelve signal crayfish individuals surpassed this threshold (single- and mixed-species stocks combined; maximum 25.32 g; Fig. S2). Signal crayfish males were heavier than females in their single stock after 45 weeks (Fig. S3).

Marbled crayfish remained the smaller species throughout the last trial with the red swamp crayfish (Fig. 1 and Fig. S1). When comparing single- and mixed-species stocks, the smaller marbled crayfish was substantially suppressed when cultured together with the red swamp crayfish, and, *vice versa*, red swamp crayfish attained on average larger sizes, albeit not statistically significant in the given species (Fig S1). The initial growth rates of both species were substantial, but decelerated around week 24, when signs of maturation were more broadly pronounced in both species (see below). At this time, the first red swamp crayfish individual achieved 30 g (Fig. S2). At 45 weeks, the red swamp crayfish individuals usually ranged between 10 and 20 g, but individuals above this were also common. On the contrary, the largest marbled crayfish attained only 7.96 g even in the single-species culture. Red swamp crayfish males were heavier than females in their single stock after 45 weeks (Fig. S3).

### Survival

**Fig. 2:**
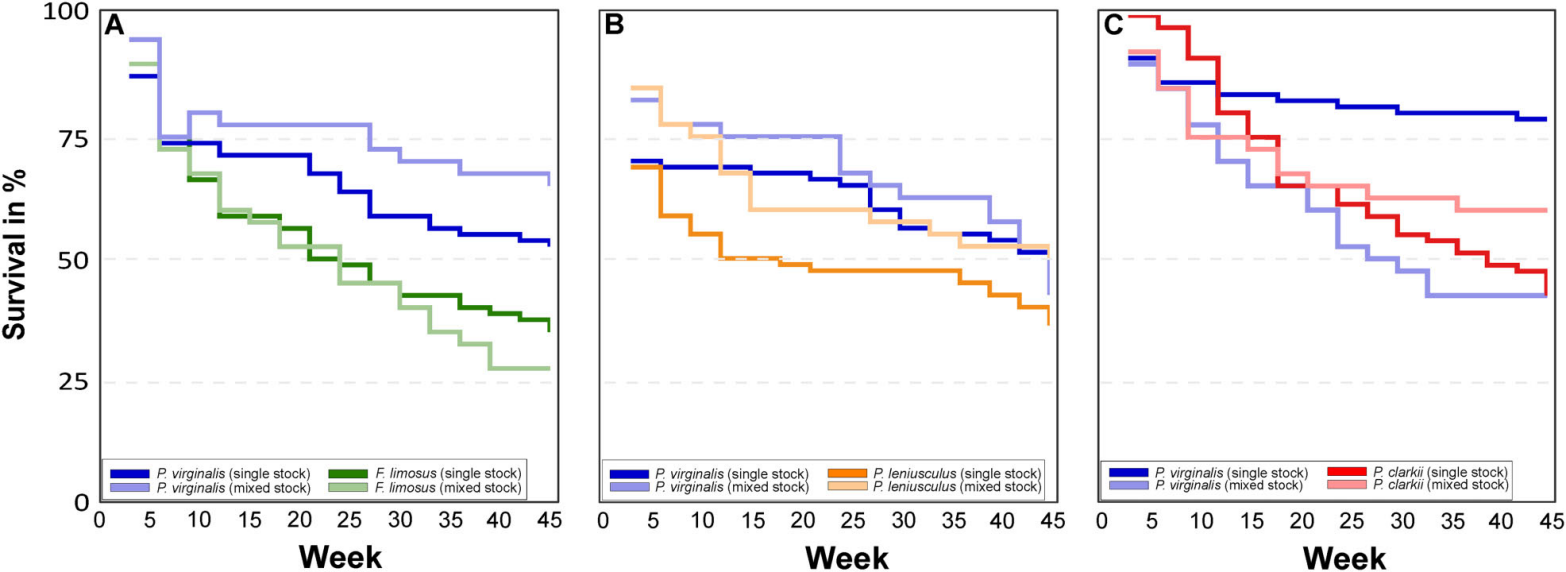
Kaplan–Meier survival curves for marbled crayfish *Procambarus virginalis* in trials with spiny-cheek crayfish *Faxonius limosus* (A), signal crayfish *Pacifastacus leniusculus* (B), and red swamp crayfish *Procambarus clarkii* (C) under single-species and mixed-species stock conditions throughout the experiment. Darker and lighter colours denote single-species and mixed-species stocks, respectively.

In the spiny-cheek crayfish trial, marbled crayfish achieved the highest survival when held in mixed stock (65.0% after 45 weeks; Fig. S4), while spiny-cheek crayfish exhibited the lowest survival in mixed stock (27.5%; Fig. 2, Table S1). Survival of marbled crayfish in single-species stock was also high (52.2%) and exceeded that of spiny-cheek crayfish in both single- (35.0%) and mixed-species stocks.

In contrast, survival curves were more balanced in the signal crayfish trial (Fig. S4 and Table S1). Signal crayfish were larger throughout the experiment, although marbled crayfish temporarily dominated intermediate growth phases (Fig. 1). The lowest survival occurred in the signal crayfish single-species stock (36.3%; Fig. 2 and Fig. S4). Final survival was similar among marbled crayfish single-species stock (51.3%), marbled crayfish mixed-species stock (42.5%), and signal crayfish mixed-species stock (50.0%).

In the red swamp crayfish trial, survival patterns resembled those of the spiny-cheek trial but under reversed size asymmetry (Fig. 2), as marbled crayfish remained substantially smaller (Fig. 1). Marbled crayfish showed the highest survival in single-species stock (78.8%), but survival declined sharply in mixed stock with red swamp crayfish (42.5%; Fig. S4 and Table S1). Red swamp crayfish also exhibited low survival in single-species stock (42.5%), significantly lower than marbled crayfish single-species survival, while mixed-stock red swamp crayfish showed intermediate survival (60.0%; Fig. S4 and Table S1).

### Reproduction

Developed glair glands were first observed in red swamp crayfish at weeks 18 and 21 in mixed- and single-species stocks, respectively (Table 2). Although male sexual development was not systematically monitored, the first reproductively mature red swamp crayfish male (Form I) was detected in the mixed-species stock during this period. In marbled crayfish, females bearing glair glands first appeared between weeks 24 (single-species stocks in the trials with spiny-cheek crayfish and red swamp crayfish) and 27 (mixed-species stock in the trial with spiny-cheek crayfish, and both stocks in the trial with signal crayfish). In these groups, the number of females with glair glands was the highest between weeks 33 and 36. Glair gland prevalence peaked between weeks 33 and 36. Marbled crayfish showed a higher probability of glair gland formation than red swamp crayfish, except in the mixed-species stock with red swamp crayfish, where the first case occurred in week 51. In spiny-cheek crayfish, the first female with glair glands was recorded after 42 weeks (mixed) and 45 weeks (single-species; Table 2).

**Table 2:** Absolute numbers of females with developed glair glands and those with attached eggs (+) recorded throughout the experiment.

	Week													
Trial	Group	18	21	24	27	30	33	36	39	42	45	48	51	54
**A**	*P. virginalis* single	–	–	2 + 0	21 + 0	26 + 0	25 + 0	31 + 1	11 + 17	7 + 6	7 + 8	3 + 7	2 + 1	2 + 4
	*F. limosus* single	–	–	–	–	–	–	–	–	–	1 + 0	NA	NA	NA
	*P. virginalis* mixed	–	–	–	13 + 0	16 + 0	17 + 0	16 + 4	5 + 13	6 + 3	2 + 5	2 + 2	1 + 2	–
	*F. limosus* mixed	–	–	–	–	–	–	–	–	1 + 0	1 + 0	NA	NA	NA
**B**	*P. virginalis* single	–	–	–	8 + 0	19 + 0	20 + 0	23 + 1	12 + 8	13 + 8	9 + 7	9 + 5	4 + 4	4 + 2 **(1)**
	*P. leniusculus* single	–	–	–	–	–	–	–	–	–	–	NA	NA	NA
	*P. virginalis* mixed	–	–	–	4 + 0	14 + 0	12 + 0	15 + 1	9 + 4	8 + 2	3 + 2	0 + 2	3 + 0	2 + 0
	*P. leniusculus* mixed	–	–	–	–	–	–	–	–	–	–	NA	NA	NA
**C**	*P. virginalis* single	–	–	4 + 0	12 + 0	20 + 0	29 + 0	21 + 1	21 + 9	16 + 15	0 + 18	12 + 21	3 + 22 **(8)**	3 + 18 **(7)**
	*P. clarkii* single	–	1 + 0	5 + 0	6 + 0	5 + 0	7 + 0	7 + 0	6 + 0	7 + 0	6 + 0	4 + 1	2 + 1	4 + 3
	*P. virginalis* mixed	–	–	–	–	–	–	–	–	–	–	–	1 + 2	1 + 3
	*P. clarkii* mixed	1 + 0	3 + 0	6 + 0	9 + 0	9 + 0	10 + 0	10 + 0	8 + 0	8 + 0	9 + 0	9 + 1	9 + 0	9 + 0

Numbers in brackets indicate ovigerous marbled crayfish that successfully hatched juveniles in some stocks at weeks 51 and 54 (highlighted in bold in brackets). Note that absolute numbers of evaluated individuals varied with the number of animals stocked and their survival (single- vs. mixed-species stocks; Fig. S4) and that marbled crayfish comprised females only. In general, we do not expect that marbled crayfish females were capable of completing two full reproductive cycles over the duration of the experiment. Data are shown for marbled crayfish *Procambarus virginalis* in trials with spiny-cheek crayfish *Faxonius limosus* (A), signal crayfish *Pacifastacus leniusculus* (B), and red swamp crayfish *Procambarus clarkii* (C).

Ovigerous marbled crayfish were first recorded at week 36 in the trials with spiny-cheek crayfish and signal crayfish across stocks (Table 2), with peak frequencies occurring shortly thereafter (week 39 in trials with spiny-cheek and signal crayfish, and later in the red swamp crayfish trial). Neither the spiny-cheek crayfish nor the signal crayfish produced eggs during the experiment. Despite frequent oviposition by marbled crayfish, no clutch hatched during the initial 45 weeks at 16°C, and many eggs subsequently showed mortality (Fig. S5). Red swamp crayfish also failed to ovulate under this temperature regime (Table 1). Following the temperature increase to 20°C, red swamp crayfish ovigerous females appeared from week 48 onward. Marbled crayfish maintained in the mixed stock with the red swamp crayfish also developed glair glands and ovulated eggs in week 51. Although none of these clutches hatched, several marbled crayfish in the single-species stock eventually carried viable hatchlings or developing juveniles in the trial with red swamp crayfish after week 51, and a single marbled crayfish female also finally hatched in the trial with signal crayfish.

## Discussion

Water temperature is a dominant driver of invasion success in aquatic ecosystems by shaping population dynamics, species interactions, and ultimately invasion trajectories of freshwater taxa (Rahel and Olden 2008). By experimentally comparing three long-established invasive crayfish species in Europe (the spiny-cheek crayfish, the signal crayfish, and the red swamp crayfish) with the newly emerging marbled crayfish under prolonged suboptimal thermal conditions (Seitz et al. 2005; Westhoff and Rosenberger 2016), this study demonstrates that low temperatures constrain absolute performance but do not necessarily eliminate competitive asymmetries among these invaders, with outcomes depending on additional ecological, biological and environmental factors.

### Growth

Despite reduced growth rates under suboptimal temperature conditions (∼16°C) compared to near-optimal environments (at ∼22°C; cf. Kouba et al. 2021), interspecific hierarchies among the tested invasive crayfish species remained consistent. Marbled crayfish rapidly overcame its smaller initial size and exceeded spiny-cheek crayfish within 3 weeks, demonstrating that their growth advantage extends beyond warm conditions (Kouba et al. 2021). This is notable given the higher thermal requirements of this species compared with spiny-cheek crayfish (Simčič et al. 2014; Faiad et al. 2023). In ecological and biogeographical studies, temperature preferences and tolerance limits are typically inferred from the range of environmental temperatures in a species’ native distribution (Zhang et al. 2020). Marbled crayfish presumably originate from Florida (Maiakovska et al. 2021), whereas spiny-cheek crayfish are native to the northeastern USA and southeastern Canada (Taylor et al. 2007), suggesting lower temperature requirements. Although the effect was modest, marbled crayfish in mixed-species stocks with spiny-cheek crayfish attained larger sizes than those in single-species cultures. Based on maximum body size alone, the species are broadly comparable, typically not exceeding 10 cm in total length, with individuals around 12 cm being rare (Hamr 2002; Chybowski 2007; Vogt 2021). Marbled crayfish consistently exhibited faster growth across both temperature regimes (Kouba et al. 2021; this study), thus conferring a competitive advantage.

Signal crayfish are native to western North America, while cryptic diversity within the species suggests a more complex phylogeographic structure than previously assumed (Taylor et al. 2007; Larson et al. 2012). The species exhibits the lowest temperature requirements, closely resembling those of generally cold-water European astacids (Westhoff and Rosenberger 2016; Viana et al. 2023). Nonetheless, marbled crayfish were able to compensate for their initially smaller size even at ∼16°C. This advantage, however, is temporary, as signal crayfish ultimately attain substantially greater size and mass, reaching a maximum of around 16 cm (Kirjavainen and Westman 1999; Buřič et al. 2021).

By contrast, as occurred at 22°C (Kouba et al. 2021), red swamp crayfish maintained a clear size advantage throughout the experiment, most pronounced in mixed-species stocks. Although native to relatively warm regions of northeastern Mexico and the southeastern USA (Taylor et al. 2007), the species exhibits substantial thermal plasticity (Westhoff and Rosenberger 2016), as further evidenced by its quasi-global distribution, including temperate regions (Oficialdegui et al. 2020; Ion et al. 2024). A previous study emphasised the temporal consistency of temperature as a crucial driver of red swamp crayfish distribution (Guareschi et al. 2024). Whereas marbled crayfish in the wild typically do not exceed 20 g, red swamp crayfish commonly reach 30–50 g due to remarkable growth rates (up to 50 g within 3–5 months; Gherardi et al. 2011), and attain total lengths of up to 15 cm (Vogt 2021). This pattern is particularly evident in males, which are generally larger in crayfish due to higher moulting frequencies and greater size increments per moult, both of which are closely linked to lower reproductive investment (Reynolds 2002).

### Survival

In accordance with Kouba et al. (2021), marbled crayfish maintained in single-species stocks consistently exhibited high survival rates (above 50% after 45 weeks of culture), suggesting relatively low intraspecific competition. This pattern may partly reflect the surplus of food and shelters provided under experimental conditions, but may also be linked to species-specific traits, including its all-female population structure and the absence of reproductive agonistic interactions, as crayfish males are generally more aggressive (Gherardi 2002). In addition, the frequent occurrence of females bearing glair glands, attached eggs, or early juveniles (Das et al. 2025), together with associated pheromonal cues (Kubec et al. 2019), may modulate aggressive interactions within the population. Finally, the comparatively small and not robust chelae of marbled crayfish (Vogt 2021) may reduce the likelihood of severe injuries during intraspecific encounters.

Nevertheless, reduced intraspecific density conferred certain advantages, as evidenced by the mixed-species stocks in which marbled crayfish were competitively dominant over spiny-cheek crayfish. Beyond size-mediated effects, survival outcomes are closely linked to behavioral interactions. Linzmaier et al. (2018) demonstrated that marbled crayfish exhibit greater aggressiveness than spiny-cheek crayfish during agonistic encounters, even when the latter are larger. Similarly, Kubec et al. (2022) reported dominance of marbled crayfish over similarly sized spiny-cheek and signal crayfish, particularly at higher temperatures (tested temperatures of 16°C, 20°C, and 24°C), from which comparable conclusions can be inferred indirectly. Chucholl et al. (2008) documented the dominance of calico crayfish *Faxonius immunis* over spiny-cheek crayfish, while Hossain et al. (2020) independently demonstrated the superiority of marbled crayfish over calico crayfish alone. Collectively, these findings suggest that behavioral dominance is a significant contributor to survival in interspecific assemblages of marbled crayfish and spiny-cheek crayfish under variable environmental conditions.

In contrast, marbled crayfish experienced substantial survival costs at sub-optimal and near-optimal temperatures when co-occurring with larger red swamp crayfish (Kouba et al. 2021; this study). Red swamp crayfish are characterised by pronounced aggressiveness, a trait that has contributed to their recognition as a globally successful invader (Souty-Grosset et al. 2016). Although marbled crayfish can be effective opponents in direct interactions, this appears to occur only when individuals are of similar size (Jimenez and Faulkes 2011; Hossain et al. 2019). Given the pronounced size disparity between the species under natural conditions, marbled crayfish are likely to be competitively disadvantaged in syntopy. A similar outcome can be expected in co-occurrence with signal crayfish, despite the marbled crayfish’s temporary early-growth advantage.

### Reproduction

From an eco-evolutionary perspective, substantial growth and high survival have limited relevance unless they ultimately translate into successful reproduction. Our experimental conditions lacked seasonal changes in temperature and natural photoperiod shifts, both of which can play specific roles in regulating crayfish reproduction (Harlıoğlu and Farhadi 2017). These cues are arguably more important for temperate species such as the signal crayfish and the spiny-cheek crayfish than for more warm-water counterparts (the marbled crayfish and the red swamp crayfish), as further discussed below. It should also be acknowledged that mimicking diel and/or seasonal temperature and light patterns may provide additional information, thereby better reflecting the situation in the wild. Also, the diet available in natural habitats is typically more diverse and may include nutrients that better support somatic growth and maturation. On the contrary, resources such as food, but also shelter, may become limited in the natural environments. Despite these limitations, the experiment still allows for informative comparisons and meaningful insights into reproductive patterns among the studied crayfish species.

First, despite the considerable duration of the experiment at 16°C and the absence of a seasonal temperature decline typical of temperate climates, maturation of signal crayfish females was not expected. Although the largest individuals reached body sizes comparable to the smallest mature individuals of the species after 45 weeks of culture, signal crayfish males generally first mature in their second year of life, with females typically maturing a year later (Lewis 2002). Importantly, successful reproduction in signal crayfish likely requires additional environmental cues, such as photoperiod shortening and temperature decline, as mating and egg attachment normally occur in autumn, followed by overwinter egg incubation and hatching in late spring or early summer (Kouba 2007; Yazicioglu et al. 2018).

In the present study, only two spiny-cheek crayfish females developed glair glands in both mixed- and single-species stocks after 43 and 45 weeks, respectively. By contrast, under warmer conditions (22°C), glair gland formation occurs substantially earlier, at approximately 13 weeks of culture (Kouba et al. 2021). These observations indicate that the experimental conditions employed were sufficient to induce glair gland development, but likely suboptimal for completing the full reproductive cycle. Under highly favourable conditions, some individuals can reproduce within their first year (Kozák et al. 2007). Spiny-cheek crayfish mate in autumn, while ovulation typically occurs the following spring, after a second peak in mating activity (Van den Brink et al. 1988; Buřič et al. 2009). This temporal pattern suggests that exposure to winter conditions serves as a critical reproductive cue. Juveniles hatch ~40–50 days post-ovulation, most commonly in June (Kozák et al. 2006). Both autumn-only and spring-only mating events are sufficient for successful reproduction, as spermatophores are retained in the female’s seminal receptacle, the *annulus ventralis*, which is a feature conserved across Cambaridae (Buřič et al. 2013). Remarkably, under experimental isolation from males, females were capable of reproducing via facultative parthenogenesis, a reproductive strategy unique among decapods, though not yet documented in natural populations (Buřič et al. 2011). Although exceedingly rare, ovulation during autumn has also been observed; however, the environmental triggers underlying such atypical timing, as well as the winter survival of early juveniles, remain poorly understood (Weiperth et al. 2020).

The red swamp crayfish is well known for its rapid growth and early sexual maturation (Souty-Grosset et al. 2016). When reared at 22°C, females bearing glair glands appeared after approximately 12 weeks of culture; however, despite frequent copulation, no oviposition occurred before experimental termination at week 15 (Kouba et al. 2021). In the present study, females with glair glands were first detected after 18 and 21 weeks in mixed-species and single-species stocks, respectively. Ovigerous females were observed only at week 45, occurring 3 weeks after water temperature was increased to 20°C. Nevertheless, no hatchlings were recorded before the experiment concluded at week 54. Collectively, these findings indicate that the experimental conditions approached the threshold required by this species to reproduce.

Females of marbled crayfish bearing glair glands were first observed at weeks 24 and 27, with ovigerous females appearing from week 34 onwards. The only exception occurred in the mixed-species treatment with red swamp crayfish, where marbled crayfish were strongly suppressed, and the first females bearing glair glands and eggs were not detected until week 51. These results demonstrate that marbled crayfish are capable of regularly forming glair glands and ovulating at 16°C, closely matching the lower reproductive threshold of 15°C reported by Seitz et al. (2005). Notably, successful hatching was, however, observed only after the temperature was increased to 20°C, occurring at weeks 51 and 54. Eggs ovulated shortly after the temperature increase also failed to develop successfully, suggesting that exposure to elevated temperatures is required during at least part of egg maturation, rather than for ovulation or early embryonic development *per se*.

Support for this interpretation comes from both laboratory and field observations. Under controlled conditions, marbled crayfish exhibit two reproductive peaks, including one in autumn (Vogt 2015). Similarly, wild populations show evidence of late-season reproduction. Lipták et al. (2017) reported numerous ovigerous females and individuals carrying attached juveniles in late October in Slovakia, while Dobrović et al. (2021) documented females with juveniles in October and November in Croatia, which is in line with observations in the Czech Republic (Buřič et al. 2025). During these periods, water temperatures frequently fall below the aforementioned thermal thresholds, yet the offspring appeared fully viable. What remains unresolved is the extent to which such early juveniles, presumably with limited energetic reserves, can survive overwintering conditions.

Under warmer conditions (22°C), the onset of marbled crayfish reproduction is substantially accelerated, with females bearing glair glands and eggs observed as early as weeks 12 and 14 after the onset of exogenous feeding (Kouba et al. 2021). To this, we should add some 2 weeks, which cover the first two mother-dependent developmental stages in this species (Guo et al. 2019). Still, this timing is earlier than previously reported (Seitz et al. 2005) and may be further shortened under more optimal conditions, such as improved dietary quality (Das et al. 2024a; 2024b). Together, these pronounced reproductive plasticities across a broad thermal range likely contribute to the exceptional invasive potential of marbled crayfish, particularly under ongoing climate warming.

## Conclusions

Our comparative experiments show that prolonged exposure to suboptimal temperatures reshapes competitive outcomes through combined effects on growth, survival, and reproductive thresholds. These patterns reflect general temperature–dependent mechanisms common across poikilothermic organisms, including shifts in behavioral dominance, changes in activity and feeding, and limits on successful recruitment. Because such mechanisms influence ecological sorting in many freshwater invaders, the crayfish system used here illustrates a broader principle: environmental filtering rarely acts uniformly and often reorganises competitive hierarchies in ways that may either promote coexistence or strengthen differences. The strongest constraints we observed were on reproduction rather than on growth or survival, which suggests that establishment under new climates is often restricted more by unsuccessful recruitment than by adult performance. As climate regimes become more variable, these processes are likely to produce complex and context–dependent invasion trajectories. Species will benefit not only from broad thermal tolerance but also from the ability to maintain competitive and reproductive function across fluctuating environmental conditions. By identifying these mechanistic pathways, our study provides a framework for predicting which invaders are most likely to persist, interact, or dominate under changing thermal conditions, in crayfish and in other temperature–sensitive freshwater organisms.

## Supplementary Material

icag014_Supplemental_Files

## Data Availability

The data underlying this article will be shared on request to the corresponding author.

## References

[bib1] Angilletta MJ Jr . 2009. Thermal Adaptation: a Theoretical and Empirical Synthesis. Oxford: Oxford University Press.

[bib2] Balzani P, Musil M, Weiperth A, Bláha M, Kubec J, Ruokonen TJ, Ercoli F, Bányai ZM, Buřič M, Veselý L et al. 2025. Seasonal changes in trophic ecology of co-occurring freshwater invasive species at a thermal locality. Hydrobiologia. 852:4493–512.

[bib3] Blackburn TM, Essl F, Evans T, Hulme PE, Jeschke JM, Kühn I, Kumschick S, Markova Z, Mrugała A, Nentwig W. 2014. A unified classification of alien species based on the magnitude of their environmental impacts. PLoS Biol. 12:e1001850. 24802715 10.1371/journal.pbio.1001850 PMC4011680

[bib4] Bláha M, Weiperth A, Patoka J, Szajbert B, Balogh ER, Staszny Á, Ferincz Á, Lente V, Maciaszek R, Kouba A. 2022. The pet trade as a source of non-native decapods: the case of crayfish and shrimps in a thermal waterbody in Hungary. Environ Monit Assess. 194:795. 36109381 10.1007/s10661-022-10361-9

[bib5] Bohman P, Edsman L, Martin P, Scholtz G. 2013. The first Marmorkrebs (Decapoda: astacida: cambaridae) in Scandinavia. BioInvasions Rec. 2:227–32.

[bib6] Bohman P, Edsman L, Sandström A, Nyström P, Stenberg M, Hertonsson P, Johansson J. 2016. Predicting harvest of non-native signal crayfish in lakes—A role for changing climate?. Can J Fish AquatSci. 73:785–92.

[bib7] Buřič M, Haubrock PJ, Veselý L, Kozák P, Kouba A. 2021. Effective investments due to seasonal morphological changes? Possible reasons and consequences of allometric growth and reproduction in adult signal crayfish (*Pacifastacus leniusculus*). Can J Zool. 99:85–96.

[bib8] Buřič M, Hulák M, Kouba A, Petrusek A, Kozák P. 2011. A successful crayfish invader is capable of facultative parthenogenesis: a novel reproductive mode in decapod crustaceans. PLoS One. 6:e20281. 21655282 10.1371/journal.pone.0020281 PMC3105005

[bib9] Buřič M, Kouba A, Kozák P. 2009. Spring mating period in *Orconectes limosus*: the reason for movement. Aquat Sci. 71:473–7.

[bib10] Buřič M, Kouba A, Kozak P. 2013. Reproductive plasticity in freshwater invader: from long-term sperm storage to parthenogenesis. PLoS One. 8:e77597. 24204886 10.1371/journal.pone.0077597 PMC3804581

[bib11] Buřič M, Ložek F, Görner T, Čuprová V, Vlach P, Kožený P, Štruncová E, Kouba A, Svobodová J. 2025. Difficult to deal with: attempts for eradication of marbled crayfish from a small urban pond. Manag Biol Invasion. 16:443–64.

[bib12] Carvalho F, Sousa R, Cássio F, Pascoal C. 2022. Temperature and interspecific competition alter the impacts of two invasive crayfish species on a key ecosystem process. Biol Invasions. 24:3757–68.

[bib13] Chucholl C, Stich HB, Maier G. 2008. Aggressive interactions and competition for shelter between a recently introduced and an established invasive crayfish: *orconectes immunis* vs. *O. limosus*. Fundam Appl Limnol. 172:27–36.

[bib14] Chybowski Ł . 2007. Morphometrics, fecundity, density, and feeding intensity of the spinycheek crayfish, *Orconectes limosus* (Raf.) in natural conditions. Fish Aquat Life. 15:175–241.

[bib15] Das K, Balzani P, Kaur D, Kubec J, Buřič M, Kouba A, Let M. 2025. Reproductive stage affects the daily behavioral patterns of parthenogenetic marbled crayfish. Curr Zool.zoaf062. 10.1093/cz/zoaf062 PMC13500160 42634740

[bib16] Das K, Roy K, Mráz J, Buřič M, Kouba A. 2024a. Considerations for fatty acids in standardized reference diet for parthenogenetic marbled crayfish *Procambarus virginalis* model organism. Sci Rep. 14:15933. 38987279 10.1038/s41598-024-66268-7 PMC11237046

[bib17] Das K, Roy K, Mráz J, Buřič M, Kouba A. 2024b. Considerations for protein and amino acids in standardized reference diet for parthenogenetic marbled crayfish *Procambarus virginalis* model organism. Sci Rep. 14:16395. 39013879 10.1038/s41598-024-58304-3 PMC11253003

[bib18] Dobrović A, Maguire I, Boban M, Grbin D, Hudina S. 2021. Reproduction dynamics of the marbled crayfish *Procambarus virginalis* Lyko, 2017 from an anthropogenic lake in northern Croatia. Aquat Invasions. 16:482–98.

[bib19] Ercoli F, Kaldre K, Paaver T, Gross R. 2019. First record of an established marbled crayfish *Procambarus virginalis* (Lyko, 2017) population in Estonia. BioInvasions Rec. 8:675–83.

[bib20] Faiad SM, Williams MA, Goodman M, Sokolow S, Olden JD, Mitchell K, Andriantsoa R, Gordon Jones JP, Andriamaro L, Ravoniarimbinina P. 2023. Temperature affects predation of schistosome-competent snails by a novel invader, the marbled crayfish *Procambarus virginalis*. PLoS One. 18:e0290615. 37703262 10.1371/journal.pone.0290615 PMC10499222

[bib21] Gherardi F . 2002. Behaviour. In: Holdich DM, editor. Biology of Freshwater Crayfish. Oxford: Blackwell Science Ltd. p.258–90.

[bib22] Gherardi F, Aquiloni L, Dieguez-Uribeondo J, Tricarico E. 2011. Managing invasive crayfish: is there a hope?. Aquat Sci. 73:185–200.

[bib23] Guareschi S, Cancellario T, Oficialdegui F, Clavero M. 2024. Insights from the past: invasion trajectory and niche trends of a global freshwater invader. Glob Change Biol. 30:e17059. 10.1111/gcb.17059 38273539

[bib24] Guo W, Kubec J, Veselý L, Hossain MS, Buřič M, McClain R, Kouba A. 2019. High air humidity is sufficient for successful egg incubation and early post-embryonic development in the marbled crayfish (*Procambarus virginalis*). Freshw Biol. 64:1603–12.

[bib25] Hamr P . 2002. Orconectes. In: Holdich DM, editor. Biology of Freshwater Crayfish. Oxford: Blackwell Science Ltd. p.585–608.

[bib26] Hamr P . 2024. The real and potential impacts of invasive crayfish in Ontario Canada: a review. Freshw Crayfish. 29:9–22.

[bib27] Harlıoğlu MM, Farhadi A. 2017. Factors affecting the reproductive efficiency in crayfish: implications for aquaculture. Aquacult Res. 48:1983–97.

[bib28] Haubrock PJ, Everts T, Abreo NAS, Bojko J, Deklerck V, Dickey JWE, Franco ACS, García-Berthou E, Katsanevakis S, Kirichenko NI et al. 2025a. The impacts of biological invasions. Biol Rev. 10.1002/brv.70124. 10.1002/brv.70124 PMC13149820 41467425

[bib29] Haubrock PJ, Kubec J, Veselý L, Buřič M, Tricarico E, Kouba A. 2019. Water temperature as a hindrance, but not limiting factor for the survival of warm water invasive crayfish introduced in cold periods. J Gt Lakes Res. 45:788–94.

[bib30] Haubrock PJ, Tarkan AS, Martín-Forés I, Katsanevakis S, Sousa R, Soto I, Green AJ, Kouba A, Everts T, Dominguez Almela V et al. 2025b. The spread of non-native species. Biol Rev. 10.1002/brv.70121. 10.1002/brv.70121 PMC13149790 41437262

[bib31] Havel JE, Kovalenko KE, Thomaz SM, Amalfitano S, Kats LB. 2015. Aquatic invasive species: challenges for the future. Hydrobiologia. 750:147–70. 32214452 10.1007/s10750-014-2166-0 PMC7087615

[bib32] Hossain MS, Guo W, Martens A, Adámek Z, Kouba A, Buřič M. 2020. Potential of marbled crayfish *Procambarus virginalis* to supplant invasive *Faxonius immunis*. Aquat Ecol. 54:45–56.

[bib33] Hossain MS, Kubec J, Kouba A, Kozák P, Buřič M. 2019. Still waters run deep: marbled crayfish dominates over red swamp crayfish in agonistic interactions. Aquat Ecol. 53:97–107.

[bib34] Hudina S, Galić N, Roessink I, Hock K. 2011. Competitive interactions between co-occurring invaders: identifying asymmetries between two invasive crayfish species. Biol Invasions. 13:1791–803.

[bib35] Ion MC, Bloomer CC, Bărăscu TI, Oficialdegui FJ, Shoobs NF, Williams BW, Scheers K, Clavero M, Grandjean F, Collas M et al. 2024. World of Crayfish™: a web platform towards real-time global mapping of freshwater crayfish and their pathogens. PeerJ. 12:e18229. 39421415 10.7717/peerj.18229 PMC11485098

[bib36] Jimenez SA, Faulkes Z. 2011. Can the parthenogenetic marbled crayfish Marmorkrebs compete with other crayfish species in fights?. J Ethol. 29:115–20.

[bib37] Kawai T, Kouba A. 2022. Postembryonic development in freshwater crayfish (Decapoda: astacidea) in an evolutionary context. Nauplius. 30:e2022001.

[bib38] Kirjavainen J, Westman K. 1999. Natural history and development of the introduced signal crayfish, *Pacifastacus leniusculus*, in a small, isolated Finnish lake, from 1968 to 1993. Aquat Living Resour. 12:387–401.

[bib39] Kordas RL, Harley CD, O’Connor MI. 2011. Community ecology in a warming world: the influence of temperature on interspecific interactions in marine systems. J Exp Mar Biol Ecol. 400:218–26.

[bib40] Kouba A . 2007. Comparison of postembryonic development of native and non-native crayfish species] [Master thesis]. University of South Bohemia in České Budějovice.

[bib41] Kouba A, Hamáčková J, Buřič M, Kozák P, 2011;Use of three forms of decapsulated *Artemia* cysts as food for juvenile noble crayfish (*Astacus astacus*). Czech J Anim Sci. 56:114–118.

[bib42] Kouba A, Lipták B, Kubec J, Bláha M, Veselý L, Haubrock PJ, Oficialdegui FJ, Niksirat H, Patoka J, Buřič M. 2021. Survival, growth, and reproduction: comparison of marbled crayfish with four prominent crayfish invaders. Biology-Basel. 10:422. 34068504 10.3390/biology10050422 PMC8151088

[bib43] Kouba A, Petrusek A, Kozák P. 2014. Continental-wide distribution of crayfish species in Europe: update and maps. Knowl Manag Aquat Ecosyst. 413:5.

[bib44] Kouba A, Tíkal J, Císař P, Veselý L, Fořt M, Příborský J, Patoka J, Buřič M. 2016. The significance of droughts for hyporheic dwellers: evidence from freshwater crayfish. Sci Rep. 6:26569. 27225308 10.1038/srep26569 PMC4880899

[bib45] Kozák P, Buřič M, Policar T. 2006. The fecundity, time of egg development and juvenile production in spiny-cheek crayfish (*Orconectes limosus*) under controlled conditions. Bull Fr Pêche Piscic. 380-381:1171–82.

[bib46] Kozák P, Buřič M, Policar T, Hamáčková J, Lepičová A. 2007. The effect of inter-and intra-specific competition on survival and growth rate of native juvenile noble crayfish *Astacus astacus* and alien spiny-cheek crayfish *Orconectes limosus*. Hydrobiologia. 590:85–94.

[bib47] Kubec J, Kouba A, Buřič M. 2019. Communication, behaviour, and decision making in crayfish: a review. Zool Anz. 278:28–37.

[bib48] Kubec J, Musil M, Krejčí M, Buřič M, Kouba A. 2022. Comparison of behavioural interactions among four invasive crayfish along a temperature gradient. Paper Presented at 22nd International Conference on Aquatic Invasive Species. Oostende, Belgium.

[bib49] Larson ER, Abbott CL, Usio N, Azuma N, Wood KA, LM HERBORG, Olden JD. 2012. The signal crayfish is not a single species: cryptic diversity and invasions in the Pacific Northwest range of *Pacifastacus leniusculus*. Freshw Biol. 57:1823–38.

[bib50] Lewis S . 2002. Pacifastacus. In: Holdich DM, editor. Biology of Freshwater Crayfish. Oxford: Blackwell Science Ltd. p.511–40.

[bib51] Linzmaier SM, Goebel LS, Ruland F, Jeschke JM. 2018. Behavioral differences in an over-invasion scenario: marbled vs. spiny-cheek crayfish. Ecosphere. 9:e02385.

[bib52] Lipták B, Liptáková P, Veselý L, Kouba A. 2018. Length frequency and morphometric analysis of the non-indigenous red-rimmed melania (*Melanoides tuberculata*) populations in Slovakia. Biologia (Bratisl). 73:505–11.

[bib53] Lipták B, Mojžišová M, Gruľa D, Christophoryová J, Jablonski D, Bláha M, Petrusek A, Kouba A. 2017. Slovak section of the Danube has its well-established breeding ground of marbled crayfish *Procambarus fallax* f. *virginalis*. Knowl Manag Aquat Ecosyst. 418:40.

[bib54] Lodge DM, Deines A, Gherardi F, Yeo DC, Arcella T, Baldridge AK, Barnes MA, Chadderton WL, Feder JL, Gantz CA et al. 2012. Global introductions of crayfishes: evaluating the impact of species invasions on ecosystem services. Annu Rev Ecol Evol Syst. 43:449–72.

[bib55] Lőkkös A, Müller T, Kovács K, Várkonyi L, Specziár A, Martin P. 2016. The alien, parthenogenetic marbled crayfish (Decapoda: cambaridae) is entering Kis-Balaton (Hungary), one of Europe’s most important wetland biotopes. Knowl Manag Aquat Ecosyst. 417:16.

[bib56] Maciaszek R, Jabłońska A, Prati S, Wróblewski P, Gruszczyńska J, Świderek W. 2022. Marbled crayfish *Procambarus virginalis* invades a nature reserve: how to stop further introductions?. Eur Zool J. 89:888–901.

[bib57] Maiakovska O, Andriantsoa R, Tönges S, Legrand C, Gutekunst J, Hanna K, Pârvulescu L, Novitsky R, Weiperth A, Sciberras A et al. 2021. Genome analysis of the monoclonal marbled crayfish reveals genetic separation over a short evolutionary timescale. Commun Biol. 4:1–7. 33462402 10.1038/s42003-020-01588-8 PMC7814009

[bib58] Mengal K, Kor G, Siino V, Levander F, Niksirat H. 2026. Effects of acute cold and heat shocks on the protein profile of crayfish hemolymph: implications for crustacean adaptation to thermal stress. Aquacult Rep. 46:103265.

[bib59] Mojžišová M, Weiperth A, Gebauer R, Laffitte M, Patoka J, Grandjean F, Kouba A, Petrusek A. 2024. Diversity and distribution of *Aphanomyces astaci* in a European hotspot of ornamental crayfish introductions. J Invertebr Pathol. 202:108040. 38081448 10.1016/j.jip.2023.108040

[bib60] O’Connor MI, Gilbert B, Brown CJ. 2011. Theoretical predictions for how temperature affects the dynamics of interacting herbivores and plants. Am Nat. 178:626–38. 22030732 10.1086/662171

[bib61] Oficialdegui FJ, Bláha M, Prati S, Lipták B, Weiperth A, Bányai ZM, Maciaszek R, Patoka J, Scheers K, Lemmers P et al. 2025. Contrasting patterns of genetic variability in pet-traded red swamp crayfish *Procambarus clarkii* and its feral populations. Freshw Biol. 70:e70008.

[bib62] Oficialdegui FJ, Sánchez MI, Clavero M. 2020. One century away from home: how the red swamp crayfish took over the world. Rev Fish Biol Fish. 30:121–35.

[bib63] O’Hea Miller SB, Davis AR, Wong MY. 2024. The impacts of invasive crayfish and other non-native species on native freshwater crayfish: a review. Biology. 13:610. 39194548 10.3390/biology13080610 PMC11351920

[bib64] Patoka J, Buřič M, Kolář V, Bláha M, Petrtýl M, Franta P, Tropek R, Kalous L, Petrusek A, Kouba A. 2016. Predictions of marbled crayfish establishment in conurbations fulfilled: evidences from the Czech Republic. Biologia (Bratisl). 71:1380–5.

[bib65] Rahel FJ, Olden JD. 2008. Assessing the effects of climate change on aquatic invasive species. Conserv Biol. 22:521–33. 18577081 10.1111/j.1523-1739.2008.00950.x

[bib66] Reynolds JD . 2002. Growth and reproduction. In: Holdich DM, editor. Biology of Freshwater Crayfish. Oxford: Blackwell Science Ltd. p.151–91.

[bib67] Šajna N, Urek T, Kušar P, Šipek M. 2023. The importance of thermally abnormal waters for Bioinvasions—A case study of *Pistia stratiotes*. Diversity. 15:421.

[bib68] Sánchez O, Oficialdegui FJ, Torralba-Burrial A, Arbesú R, Valle-Artaza JM, Fernández-González Á, Ardura A, Arias A 2024. *Procambarus virginalis* Lyko, 2017: a new threat to Iberian inland waters. Ecol Evol. 14:e11362. 38774140 10.1002/ece3.11362 PMC11106043

[bib69] Sayer CA, Fernando E, Jimenez RR, Macfarlane NB, Rapacciuolo G, Böhm M, Brooks TM, Contreras-MacBeath T, Cox NA, Harrison I. 2025. One-quarter of freshwater fauna threatened with extinction. Nature. 638:138–45. 39779863 10.1038/s41586-024-08375-z PMC11798842

[bib70] Seitz R, Vilpoux K, Hopp U, Harzsch S, Maier G. 2005. Ontogeny of the Marmorkrebs (marbled crayfish): a parthenogenetic crayfish with unknown origin and phylogenetic position. J Exp Zool Part A. 303:393–405. 10.1002/jez.a.143 15828010

[bib71] Simčič T, Pajk F, Jaklič M, Brancelj A, Vrezec A. 2014. The thermal tolerance of crayfish could be estimated from respiratory electron transport system activity. J Therm Biol. 41:21–30. 24679968 10.1016/j.jtherbio.2013.06.003

[bib72] Soto I, Balzani P, Carneiro L, Cuthbert RN, Macêdo R, Serhan Tarkan A, Ahmed DA, Bang A, Bacela-Spychalska K, Bailey SA et al. 2024. Taming the terminological tempest in invasion science. Biol Rev. 99:1357–90. 38500298 10.1111/brv.13071

[bib73] Souty-Grosset C, Anastácio PM, Aquiloni L, Banha F, Choquer J, Chucholl C, Tricarico E. 2016. The red swamp crayfish *Procambarus clarkii* in Europe: impacts on aquatic ecosystems and human well-being. Limnologica. 58:78–93.

[bib74] Takács P, Bánó B, Czeglédi I, Pallos R, Erős T, Weiperth A. 2025. Alien fishes in Hungary: the rise of aquarium species. Biol Invasions. 27:166.

[bib75] Taylor CA, Schuster GA, Cooper JE, DiStefano RJ, Eversole AG, Hamr P, Hobbs HH III, Robison HW, Skelton CE, Thoma RE. 2007. Feature: endangered species—A reassessment of the conservation status of crayfishes of the united states and Canada after 10+years of increased awareness. Fisheries. 32:372–89.

[bib76] Therneau TM, Grambsch PM. 2000. The Cox Model. Modeling Survival Data: Extending the Cox Model. Springer. p.39–77.

[bib77] Urban MC, Bocedi G, Hendry AP, Mihoub J-B, Pe’er G, Singer A, Bridle JR, Crozier LG, De Meester L, Godsoe W. 2016. Improving the forecast for biodiversity under climate change. Science. 353:aad8466. 27609898 10.1126/science.aad8466

[bib78] Van den Brink F, Van der Velde G, Geelen J. 1988. Life history parameters and temperature-related activity of an American crayfish, *Orconectes limosus* (Rafinesque, 1817) (Crustacea, Decapoda), in the area of the major rivers in The Netherlands. Arch Hydrobiol. 114:275–89.

[bib79] Veselý L, Buřič M, Kouba A. 2015. Hardy exotics species in temperate zone: can “warm water” crayfish invaders establish regardless of low temperatures?. Sci Rep. 5:16340. 26572317 10.1038/srep16340 PMC4648075

[bib80] Veselý L, Hrbek V, Kozák P, Buřič M, Sousa R, Kouba A, 2017;Salinity tolerance of marbled crayfish *Procambarus fallax* f. *virginalis*. Knowl Manag Aquat Ecosys. 418:21.

[bib81] Viana DS, Oficialdegui FJ, Soriano MC, Hermoso V, Clavero M. 2023. Niche dynamics along two centuries of multiple crayfish invasions. J Anim Ecol. 92:2138–50. 37731343 10.1111/1365-2656.14007

[bib82] Vogt G . 2015. Bimodal annual reproductive pattern in laboratory-reared marbled crayfish. Invertebr Reprod Dev. 59:218–23.

[bib83] Vogt G . 2021. Evaluation of the suitability of the parthenogenetic marbled crayfish for aquaculture: potential benefits versus conservation concerns. Hydrobiologia. 848:285–98.

[bib84] Weiperth A, Bláha M, Szajbert B, Seprős R, Bányai Z, Patoka J, Kouba A. 2020. Hungary: a European hotspot of non-native crayfish biodiversity. Knowl Manag Aquat Ecosyst. 421:43.

[bib85] Westhoff JT, Rosenberger AE. 2016. A global review of freshwater crayfish temperature tolerance, preference, and optimal growth. Rev Fish Biol Fish. 26:329–49.

[bib86] Yazicioglu B, Kouba A, Kozák P, Niksirat H. 2018. Post-mating spermatophore storage strategies in two species of crayfish: implications for broodstock management. Animal. 12:554–8. 28747233 10.1017/S1751731117001744

[bib87] Zhang Z, Capinha C, Usio N, Weterings R, Liu X, Li Y, Landeria JM, Zhou Q, Yokota M. 2020. Impacts of climate change on the global potential distribution of two notorious invasive crayfishes. Freshw Biol. 65:353–65.

