# Supplementary material for: Life History Responses of Four Invasive Crayfish Species Under Prolonged Suboptimal Temperatures
Source: Integr Comp Biol. 2026 Mar 23;66:icag014. doi: 10.1093/icb/icag014 (PMC13069686; doi:10.1093/icb/icag014)
Supplement: icag014_Supplemental_Files [file icag014_supplemental_files.zip › icb-2026-0016-File002.docx]

**Life history responses of four invasive crayfish species under prolonged suboptimal temperatures**

**Running title:** Crayfish at suboptimal temperatures

Antonín Kouba^1,*^, Koushik Das^1,2^, Wei Guo^1,3^, Kateřina Marková^1^, Lukáš Veselý^1^, Francisco J. Oficialdegui^1,4^, Boris Lipták^1,5^, Jan Kubec^1^, Anna Koubová^1^, Martin Bláha^1^, Hamid Niksirat^1^, Jiří Patoka^6,7,8^, András Weiperth^9,10^, Phillip J. Haubrock^1,11,#^, Miloš Buřič^1,#^

^1^ *Faculty of Fisheries and Protection of Waters, South Bohemian Research Center of Aquaculture and Biodiversity of Hydrocenoses, University of South Bohemia in České Budějovice, Zátiší 728/II, 389 01 Vodňany, Czech Republi*c

^2^ College of Biosystems Engineering and Food Science, Zhejiang University, 310000 Hangzhou, China.

^3^ *Xinjiang Key Laboratory for Ecological Adaptation and Evolution of Extreme Environment Organisms, College of Life Sciences, Xinjiang Agricultural University, Nongda East Road No. 311, Shayibak District, Urumqi 830000, China*

^4^ *Department of Conservation Biology and Global Change, Doñana Biological Station (CSIC), C/Americo Vespucio 26, 41092, Seville, Spain*

*^5^ Slovak Environment Agency, Tajovského 28, 97590 Banská Bystrica, Slovak Republic*

^6^ *Department of Zoology and Fisheries, Faculty of Agrobiology, Food and Natural Resources, Czech University of Life Sciences Prague, Kamýcká 129, 16500 Prague-Suchdol, Czech Republic*

^7^ *Department of Preschool & Primary Education, Faculty of Education, Jan Evangelista Purkyně University in Ústí nad Labem, České mládeže 8, 400 01 Ústí nad Labem, Czech Republic*

^8^ *Department of Biology, Faculty of Science, Humanities and Education, Technical University of Liberec, Studentská 1402/2, 461 17 Liberec, Czech Republic*

*^9^ Department of Systematic Zoology and Ecology, Institute of Biology, ELTE Eötvös Loránd University, Pázmány Péter ave 1/C, H-1117 Budapest, Hungary*

*^10^ Dr. Puky Miklós Toad Action Group, Endrődi Sándor street. 85/A, H-1026 Budapest, Hungary*

^11^ *Department of Life and Environmental Sciences, Bournemouth University, Poole, Dorset, Talbot Campus, Fern Barrow, Poole, Dorset, BH12 5BB, UK4*

^*^Corresponding author e-mail: [akouba@frov.jcu.cz](mailto:akouba@frov.jcu.cz)

^#^ These authors contributed equally

**Abstract**

Biological invasions are strongly shaped by temperature, especially in poikilothermic organisms, where thermal regimes influence life-history traits, thereby determining both their competitive potential and geographic distribution. However, comparative evidence on how suboptimal thermal conditions modulate interactions among co-occurring invasive species remains scarce. We experimentally compared the growth, survival, and reproductive performance of the invasive parthenogenetic marbled crayfish *Procambarus virginalis* with three widespread North American crayfish invaders in Europe: the spiny-cheek crayfish *Faxonius limosus*, the signal crayfish *Pacifastacus leniusculus*, and the red swamp crayfish *Procambarus clarkii*. Experiments were conducted under prolonged suboptimal conditions (~16 °C over 45 weeks), followed by a short-term temperature increase (~20 °C). Across three independent laboratory trials, we assessed species performance in single-species and mixed-species stocks. Despite reduced absolute growth rates at low temperature, marbled crayfish rapidly compensated for their initially smaller size and outperformed spiny-cheek crayfish in growth and survival. In contrast, marbled crayfish were consistently suppressed when co-occurring with the larger and more aggressive red swamp crayfish, whereas interactions with signal crayfish resulted in temporary growth advantages but ultimately size convergence. Survival patterns reflected a combination of size asymmetries, behavioural dominance, and intraspecific aggression, with marbled crayfish exhibiting notably high survival in single-species stocks across all trials. Reproductive development was strongly temperature-constrained. While marbled crayfish readily formed glair glands and ovulated eggs at 16 °C, successful hatching occurred only after the temperature was raised. Our results demonstrate that suboptimal thermal conditions do not eliminate competitive asymmetries among invasive crayfish but instead reshape invasion outcomes in species-specific ways. These findings highlight the marbled crayfish's capacity to persist and interact competitively even in colder environments, with important implications for invasion dynamics under ongoing climate change.

**Keywords:** *biological invasion; pet trade; animal release; species interactions; syntopy; sympatry*
