# Supplementary material for: Life History Responses of Four Invasive Crayfish Species Under Prolonged Suboptimal Temperatures
Source: Integr Comp Biol. 2026 Mar 23;66:icag014. doi: 10.1093/icb/icag014 (PMC13069686; doi:10.1093/icb/icag014)
Supplement: icag014_Supplemental_Files [file icag014_supplemental_files.zip › icb-2026-0016-File008.doc]

**Life history responses of four invasive crayfish species under prolonged suboptimal temperatures**

Antonín Kouba^1,*^, Koushik Das^1,2^, Wei Guo^1,3^, Kateřina Marková^1^, Lukáš Veselý^1^, Francisco J. Oficialdegui^1,4^, Boris Lipták^1,5^, Jan Kubec^1^, Anna Koubová^1^, Martin Bláha^1^, Hamid Niksirat^1^, Jiří Patoka^6,7,8^, András Weiperth^9,10^, Phillip J. Haubrock^1,11,#^, Miloš Buřič^1,#^

^1^ *Faculty of Fisheries and Protection of Waters, South Bohemian Research Center of Aquaculture and Biodiversity of Hydrocenoses, University of South Bohemia in České Budějovice, Zátiší 728/II, 389 01 Vodňany, Czech Republi*c

^2^ College of Biosystems Engineering and Food Science, Zhejiang University, 310000 Hangzhou, China.

^3^ *Xinjiang Key Laboratory for Ecological Adaptation and Evolution of Extreme Environment Organisms, College of Life Sciences, Xinjiang Agricultural University, Nongda East Road No. 311, Shayibak District, Urumqi 830000, China*

^4^ *Department of Conservation Biology and Global Change, Doñana Biological Station (CSIC), C/Americo Vespucio 26, 41092, Seville, Spain*

*^5^ Slovak Environment Agency, Tajovského 28, 97590 Banská Bystrica, Slovak Republic*

^6^ *Department of Zoology and Fisheries, Faculty of Agrobiology, Food and Natural Resources, Czech University of Life Sciences Prague, Kamýcká 129, 16500 Prague-Suchdol, Czech Republic*

^7^ *Department of Preschool & Primary Education, Faculty of Education, Jan Evangelista Purkyně University in Ústí nad Labem, České mládeže 8, 400 01 Ústí nad Labem, Czech Republic*

^8^ *Department of Biology, Faculty of Science, Humanities and Education, Technical University of Liberec, Studentská 1402/2, 461 17 Liberec, Czech Republic*

*^9^ Department of Systematic Zoology and Ecology, Institute of Biology, ELTE Eötvös Loránd University, Pázmány Péter ave 1/C, H-1117 Budapest, Hungary*

*^10^ Dr. Puky Miklós Toad Action Group, Endrődi Sándor street. 85/A, H-1026 Budapest, Hungary*

^11^ *Department of Life and Environmental Sciences, Bournemouth University, Poole, Dorset, Talbot Campus, Fern Barrow, Poole, Dorset, BH12 5BB, UK4*

^*^Corresponding author e-mail: [akouba@frov.jcu.cz](mailto:akouba@frov.jcu.cz)

^#^ These authors contributed equally

**Figure S1.** Weight (g) of marbled crayfish *Procambarus virginalis* in trials with spiny-cheek crayfish *Faxonius limosus* (A), signal crayfish *Pacifastacus leniusculus* (B), and red swamp crayfish *Procambarus clarkii* (C) under single-species and mixed-species stock conditions throughout the experiment. Darker and lighter colours denote single-species and mixed-species stocks, respectively. Where applicable, different superscript letters indicate statistically significant differences among groups within a given week (Kruskal–Wallis test followed by multiple comparisons of mean ranks; p < 0.05). Data are presented as mean and SD. Note the differing y-axis scales among trials.

**Figure S2.** Weight (g) of marbled crayfish *Procambarus virginalis* in trials with spiny-cheek crayfish *Faxonius limosus* (A), signal crayfish *Pacifastacus leniusculus* (B), and red swamp crayfish *Procambarus clarkii* (C) under single-species and mixed-species stock conditions throughout the experiment. Darker and lighter colours denote single-species and mixed-species stocks, respectively. Note the differing y-axis scales among trials and relative to Fig. 1, which were adjusted to better depict the observed intraspecific variability.

**Figure S3.** Sex-specific weight (g) in sexually reproducing species—spiny-cheek crayfish *Faxonius limosus* (A), signal crayfish *Pacifastacus leniusculus* (B), and red swamp crayfish *Procambarus clarkii* (C)—maintained under single-species and mixed-species stock conditions after 45 weeks of culture. Asterisks (*) indicate statistically significant differences between sexes within a given stock (Mann–Whitney U test, p < 0.05). Data are presented as mean and SD.

**Figure S4.** Marbled crayfish *Procambarus virginalis* in trials with spiny-cheek crayfish *Faxonius limosus* (A), signal crayfish *Pacifastacus leniusculus* (B), and red swamp crayfish *Procambarus clarkii* (C) under single-species and mixed-species stock conditions throughout the experiment. Darker and lighter colours denote single-species and mixed-species stocks, respectively. Where applicable, different superscript letters indicate statistically significant differences among groups within a given week (percent survival arc-sine transformed, Kruskal–Wallis test followed by multiple comparisons of mean ranks; p < 0.05). Data are presented as mean and SD.

**Table S1.** Output of the Kaplan–Meier survival analysis for marbled crayfish *Procambarus virginalis* in trials with spiny-cheek crayfish *Faxonius limosus* (A), signal crayfish *Pacifastacus leniusculus* (B), and red swamp crayfish *Procambarus clarkii* (C) maintained under single-species and mixed-species stock conditions throughout the experiment. Statistically significant values are highlighted in bold.

| **Trial** | **Test** | **Chisq** | **df** | **p** |
| --- | --- | --- | --- | --- |
| A | Overall | 17.3 | 3 | **< 0.001** |
|  | *P. virginalis* single vs *F. limosus* single | 4.9 | 1 | **0.03** |
|  | *P. virginalis* single vs *F. limosus* mixed | 4.9 | 1 | **0.03** |
|  | *P. virginalis* mixed vs *F. limosus* single | 0.1 | 1 | 0.8 |
|  | *P. virginalis* mixed vs *F. limosus* mixed | 12.4 | 1 | **< 0.001** |
|  | *P. virginalis* single vs *P. virginalis* mixed | 3.1 | 1 | 0.08 |
|  | *F. limosus* single vs *F. limosus* mixed | 0.1 | 1 | 0.8 |
| B | Overall | 5.1 | 3 | 0.2 |
| C | Overall | 21.7 | 3 | **< 0.001** |
|  | *P. virginalis* single vs *P. clarkii* single | 20.2 | 1 | **< 0.001** |
|  | *P. virginalis* single vs *P. clarkii* mixed | 3.4 | 1 | 0.07 |
|  | *P. virginalis* mixed vs *P. clarkii* single | 0 | 1 | 0.9 |
|  | *P. virginalis* mixed vs *P. clarkii* mixed | 1.9 | 1 | 0.2 |
|  | *P. virginalis* single vs *P. virginalis* mixed | 12.7 | 1 | **< 0.001** |
|  | *P. clarkii* single vs *P. clarkii* mixed | 3.2 | 1 | 0.07 |

**Figure S5.** Ovigerous marbled crayfish *Procambarus virginalis* maintained at 16 °C. In some females, a portion or all eggs were already dead (orange) at ovulation (note the glair still present on the abdomen, and glair glands most visible on the uropods; A). In others, at least some eggs appeared viable (grey; B). Despite this, no females successfully reproduced under these temperature conditions (C).
